# Spontaneous Pneumothorax as a Complication of COVID-19 Pneumonia: A Case Report

**DOI:** 10.5811/cpcem.2020.8.49139

**Published:** 2020-09-08

**Authors:** Leland Perice, Zhanna Roit, Ingrid Llovera, Mary G. Flanagan-Kundle

**Affiliations:** * North Shore University Hospital – Northwell Health, Department of Emergency Medicine, Manhasset, New York; † Touro College School of Health Sciences, Physician Assistant Program, Bay Shore, New York

**Keywords:** COVID-19, tension pneumothorax, necrotizing pneumonia, point-of-care ultrasound

## Abstract

**Introduction:**

Coronavirus disease 2019 (COVID-19) is caused by the severe acute respiratory syndrome coronavirus 2. It typically presents with respiratory symptoms such as fevers, cough, and shortness of breath. As the number of cases increases, however, COVID-19 is being increasingly recognized as being associated with a variety of other respiratory pathologies.

**Case Report:**

We present the case of a 59-year-old man with COVID-19 pneumonia who acutely decompensated after having been on the medicine floor for two weeks. He was found to have a tension pneumothorax. This was treated with a needle decompression followed by a chest tube insertion. The patient subsequently recovered and was discharged.

**Conclusion:**

This case highlights the importance of considering tension pneumothorax as a possible cause of shortness of breath in patients with COVID-19 pneumonia.

## INTRODUCTION

On March 11, 2020, coronavirus disease 2019 (COVID-19) was declared a global pandemic by the World Health Organization after it was identified in Wuhan, China in December 2019. As of September 1, 2020 there have been 169,419 deaths nationally.^1^ The symptoms of COVID-19 typically include shortness of breath, dry cough, fever, fatigue, muscle aches, and sore throat. There is an incubation period of 1–14 days before the onset of symptoms.^2^ In more severe cases, patients are hypoxic and develop acute respiratory failure, requiring intensive care and mechanical ventilatory support. Many of these patients have radiographic findings of bilateral lung, ground-glass opacities.^3^

In the context of a pandemic, many healthcare institutions have created protocols to efficiently address the most common presentations of this disease. Yet despite often presenting with similar symptoms, it is important to keep a wide differential when treating these patients as COVID-19 has been linked to a variety of pathologies. Reported complications secondary to COVID-19 include myocarditis, pulmonary embolism, large vessel cerebral vascular accidents, and acute respiratory distress syndrome.^4^ Here we present a patient who initially presented to the emergency department (ED) with shortness of breath and hypoxia, and while an inpatient rapidly decompensated secondary to a spontaneous tension pneumothorax.

## CASE REPORT

A 59-year-old male with no past medical history presented to the ED with cough, fevers, and shortness of breath. He was found to be hypoxic requiring 15 liters of oxygen on a non-rebreather mask to maintain an oxygen saturation above 90% on pulse oximetry. Computed tomography (CT) angiogram of the chest performed in the ED found pulmonary emboli within the segmental branches of the right upper and both lower lobe pulmonary arteries along with patchy areas of ground-glass opacities and consolidation. Polymerase chain reaction tests of nasal and pharyngeal swabs and sputum performed in the ED were positive for severe acute respiratory syndrome coronavirus 2 RNA. He was started on a therapeutic dose of enoxaparin and then admitted to the medicine floor for further treatment.

**Image 1:**
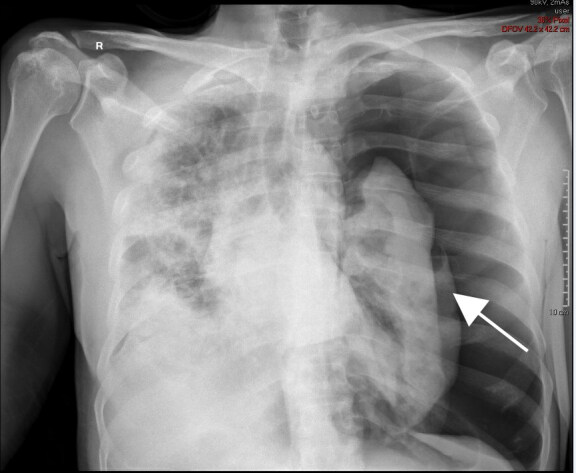
Tension pneumothorax of the left lung on chest radiograph. Arrow indicates compressed left lung surrounded by free air.

His respiratory status improved and he was eventually weaned to one liter of oxygen via nasal cannula by day 10 of hospitalization. On day 11 of hospitalization the patient had a sudden desaturation to 60% on pulse oximetry. The rapid response team was called, and a portable chest radiograph (CXR) was performed, which found a large pneumothorax (Image 1).

**Image 2:**
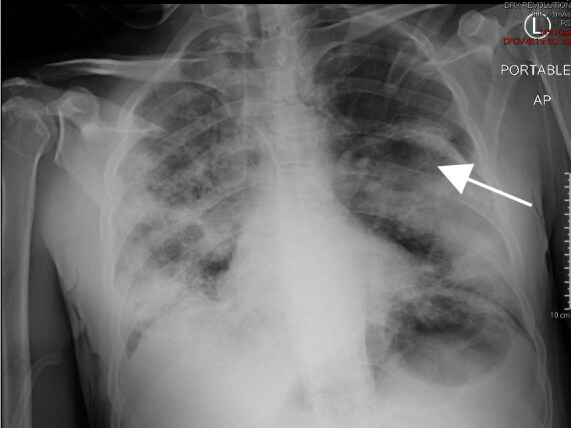
Tension pneumothorax improved after chest tube placement. Arrow indicates area of re-expansion.

Emergent needle decompression of the tension pneumothorax with an 18-gauge needle and subsequent 10 French chest tube placement were immediately performed. The patient had rapid clinical improvement after needle decompression with improvement of his oxygen saturation to 100% while on 15 liters of oxygen through a non-rebreather mask and re-expansion of his lung on CXR (Image 2).

On day 18 of hospitalization the patient’s hemoglobin dropped from 9.1 grams (g) per deciliter (dL) to 7.2 g/dL. Repeat CT was subsequently performed and showed bilateral loculated hemopneumothoraces. The patient was started on vancomycin and piperacillin/tazobactam due to concern for necrotizing pneumonia as the underlying cause of the new lung changes, and his anticoagulation was switched from therapeutic enoxaparin injections to a heparin infusion. He continued to clinically improve, and the chest tube was removed. He was eventually discharged from the hospital on apixaban, doxycycline hyclate, and amoxicillin/clavulanate potassium.

CPC-EM Capsule

What do we already know about this clinical entity?

*Coronavirus disease 2019 (COVID-19) is known to present with respiratory symptoms such as cough, fevers, and shortness of breath*.

What makes this presentation of disease reportable?

*Pneumothorax is not a known complication of COVID-19. Here we present a patient whose pneumothorax occurred as a direct complication of COVID-19*.

What is the major learning point?

*Pneumothorax should be in the differential diagnosis of patients with COVID-19 who present with shortness of breath*.

How might this improve emergency medicine practice?

*Patients with COVID-19 and shortness of breath should have early evaluation with a chest radiograph or point-of-care ultrasound*.

## DISCUSSION

We present a case of a healthy 59-year-old male, admitted to the hospital for COVID-19 pneumonia, who acutely decompensated secondary to a tension pneumothorax after having improved for 11 days on the medical floor. He had been improving from COVID-19 for three weeks prior to deteriorating. The patient subsequently improved with chest thoracostomy and antibiotics.

Although the pneumothorax cannot definitively be considered a complication of COVID-19, our patient otherwise had no risk factors for developing a spontaneous pneumothorax. Multiple case reports from other sources have also documented spontaneous pneumothoraxes in healthy patients with COVID-19.^5^–^6^ In these other case reports the pneumothoraxes occurred many weeks into the course of the illness as in the case of our patient. This is also consistent with the evolution of radiological lung findings described in a patient from Wuhan, China, who was critically ill with COVID-19 pneumonia.^7^ The initial ground-glass opacities progress to consolidations and then within weeks eventually develop into large bullae. It is possible that these bullae rupture with increases in intrathoracic pressure, such as from coughing, resulting in a pneumothorax. This is a possible mechanism by which a pneumothorax can be a direct complication of COVID-19 pneumonia in patients who otherwise have no specific risk factors for pneumothorax. It is also possible that these bullae result in bleeding, as seen in our patient, or become superinfected.

COVID-19 often presents with hypoxia and shortness of breath with the diagnosis of pneumonia. Early mechanical ventilation has been suggested for acute hypoxemic respiratory failure in the context of COVID-19.^8^–^9^ However, this case demonstrates the importance of considering other diagnoses, keeping a broad differential in mind, and not anchoring on the most common COVID-19-related clinical sequelae. It is important that the clinician perform a thorough clinical exam to assess whether there are complications such as a tension pneumothorax. It is easily reversible but if left undiagnosed can cause rapid deterioration and death. If our patient had empirically been placed on mechanical ventilation with a tension pneumothorax, the clinical outcome would likely have been worse.

Patients with COVID-19 should all have early radiological evaluation and repeat evaluation during any decompensation to evaluate for pneumothorax as the underlying cause of decompensation. This case highlights the importance of point-of-care ultrasound, which could have found the tension pneumothorax sooner and resulted in earlier placement of the chest tube. Making the diagnosis of a tension pneumothorax quickly is essential to early, lifesaving intervention.

## CONCLUSION

The emergency physician should include pneumothorax in the differential diagnosis of suspected COVID patients who present with shortness of breath. Tension pneumothorax is an easily reversible cause of shortness of breath but, if untreated, can be lethal.
